# Death of the genome paper

**DOI:** 10.3389/fgene.2013.00072

**Published:** 2013-05-06

**Authors:** David Roy Smith

**Affiliations:** Department of Biology, Western UniversityLondon, ON, Canada

There is a bloated folder on my laptop computer called *Limbolandomics*, and stuffed inside it are dozens of genome sequences eagerly waiting to be analyzed, written up, and published. I haven't told the sequences yet, but their chance to shine in the academic spotlight, to have their nucleotides forever inscribed in the annals of scientific literature, even in an obscure journal, may never come. The “genome paper” is dying and could soon be dead.

Not long ago, a folder full of genomes would have been worth its weight in high-impact publications. The formula was simple. Open with a catchy introduction about the organism of interest, emphasizing its broad biological importance and the many questions that its DNA sequence will help answer, then describe the genome in all its glory, using beautiful chromosome maps and dizzying Venn diagrams.

Genome papers have been the bread and butter of evolutionary biologists and geneticists for decades. They've been cultivated, packaged, promoted, and used to describe just about every type of DNA molecule imaginable. From the itty-bitty genomes of viruses, plasmids, and organelles to the gargantuan nuclear genomes of plants, animals, and protists, from the oh-so-boring-won't-reveal-a-darn-thing-about-anything genomes to the super-cool-send-me-to-*Nature* genomes, you name it, there's a genome paper for it.

I built my PhD thesis on genome papers, and most of my peers did the same. My first undergraduate research project was to sequence and describe the mitochondrial genome of the giant sea scallop. On top of being a riveting conversation starter at the campus pub, the scallop genome taught me tons about genetics and the publication process. A genome project, when the sequence is manageable, like a mitochondrial DNA, can be an excellent teaching tool. There is a well-defined goal, the methods are generally straightforward and cover a wide range of techniques, from molecular biology to population genetics to bioinformatics, and writing up the data is often enjoyable, and in some ways similar to a character sketch.

One of the drawbacks of genome papers, however, is that they can create a mindset of sequence first, ask questions later. I once attended a Masters thesis defense where the external examiner asked the candidate why he sequenced the chloroplast genome of this particular species and what hypothesis was he trying to test. The student, looking startled, answered, “Because the genome hadn't been sequenced before and we didn't know what it looked like.” After the defense, I overheard the examiner in the hallway venting to another professor. “We've created a culture of serial genomicists,” she exclaimed. “Everyone's jumping from one genome sequence to the next, looking to score a major publication.”

Regardless of this opinion, genome papers have provided much of the raw data that have shaped our view of genetics and evolution over the past 20 years. And they can also be a joy to read. Many of my favorite journal articles are genome papers. I remember, when I was a grad student in phycology, eagerly awaiting publication of the genome for *Chlamydomonas*—the superstar of green algae—and reading it incessantly once it was released, gleaning new insights each time through. There is something intimate and personal in learning about a species' genome. And similarly, if you are part of the team describing the genome, there is a feeling that you're giving the readers a first glimpse at an uncharted territory, with its unique landscape of genes, introns and intergenic regions.

But all of this may be coming to an end. Next generation DNA sequencing techniques have made it easy, fast and cheap to sequence genomes. Today, just about any scientist can walk out their laboratory doors, point to a living thing and say, “I will sequence you!” High-throughput technologies have flooded the academic market with genome papers. And the top journals have responded by only accepting papers describing the most novel, earth-shattering genomes. The less spectacular genomes, much like B-movies, go directly to video, or rather directly to GenBank. This sequencing-vs-publishing arms race has been going on for a long time.

Once during my PhD I whined to my supervisor about having to wait a week for the results of some PCR products that we had sent for Sanger sequencing. Professor Lee gave me a disapproving look and said, “Come with me.” We walked to his jumbled office where he shuffled through an old rickety filing cabinet, eventually pulling out a dusty autoradiogram of a dideoxy sequencing gel. “Do you know what this is, Smitty?” he said, waving the autoradiogram above his head like a holy tablet of sequencing. “Do you realize that it took me eight painful months to get this short stretch of sequence?” His point was made: sequencing DNA was once bloody hard and intensely boring work. But, as my supervisor can attest, just a little bit of sequence data was enough for a PhD and a *PNAS* paper.

The traditional genome paper will surely evolve into new, more complex forms. This has already started to happen. Now, it is not uncommon to see publications that combine multiple genomes into a single paper or ones that present a genome combined with a transcriptome and a proteome. In some journals, genome sequences have been relegated to a special category of paper called “Genome Reports” or “Genome Announcements,” which are highly reduced, sometimes containing fewer than 500 words. But no matter if the changes are for the better or worse, many of us will lament the passing of the original form.

Is it time to write the genome paper obituary? Maybe not quite yet. Every now and then they still claw their way into top journals. But the end is not far off, and when it does come, I'm sure that I speak for all of us genome geeks when I say, “Farewell, GP. It was fun while it lasted.”

